# The Study of Sexual Satisfaction in Iranian Women Applying for Divorce

**Published:** 2014-11-01

**Authors:** Farzad Gheshlaghi, Gholamali Dorvashi, Farzaneh Aran, Faranak Shafiei, Gita Montazeri Najafabadi

**Affiliations:** 1 Department of Clinical Toxicology, School of Medicine, Isfahan University of Medical Sciences, Isfahan, Iran; 2 Isfahan Clinical Toxicology Research Center, Isfahan University of Medical Sciences, Isfahan, Iran

**Keywords:** Sexual, Divorce, Women

## Abstract

**Background:**

Marital instability is affected by many factors. In Iran, socio-cultural and political limitations are obstacles for sexuality-related studies; therefore, insufficient in- formation is available in this area. In the present research, we investigated the relation- ship between marital instability and sexual satisfaction among Iranian women.

**Materials and Methods:**

A case-control study was carried out to investigate women ap- plying for divorce in comparison with our controls during 2011 to 2012 in Isfahan, Iran. Data gathering was done using a questionnaire including two parts: socio-demographic information and factors influencing sexual satisfaction. Larson Inventory of Sexual Sat- isfaction for determining sexual satisfaction was used to determine sexual satisfaction.

**Results:**

Divorce rate is significantly related to sexual satisfaction (p=0.009). There were also significant relationships between sexual satisfaction and the following variables: age, economic status, amount of income, duration of marriage, number of children, hous- ing, alcohol/drug abuse by spouse, being beaten by spouse, compulsory marriage, second marriage of spouse, and being happy with current partner.

**Conclusion:**

Sexual satisfaction plays an important role in marital stability of Iranian women. Thus, development of practical strategies in order to provide cultural intervention is needed to improve Iranian couples’ awareness of their sexual relationship. Indeed, train- ings in communication skills through sexual encounters are essential.

## Introduction

Sexuality is an important part of the whole person, while is considered as integral component of health and general well-being in order to have better quality of life (1, 2). Sexuality affects individual’s social life by influencing his/her behavior toward himself/herself, his/her sexual partner, and all other people (1, 3). Satisfaction of these sexual desires has a determinant role in order to form the human personality, while separation of these desires from any human behavior is inevitable (3).

Sexual satisfaction as a pleasurable feeling resulting from individual behaviors and interpersonal interactions is defined as judgment and analysis of one’s own sexual behavior that is unlike some sources considering sexual satisfaction as a means of "orgasm" (4).

Sexual satisfaction is one of the necessities for a strong and sustainable marital relationship, and is correlated with mental health, general happiness, professional achievements and successful social interactions (5).

Sexual satisfaction is affected by different factors like job stress, couples’ struggles, education level, cultural influences, economic problems, moral and sexual consistency, and physical and mental diseases (3).

Sexual dissatisfaction leads to negative mental and spiritual effects, like disappointment, depression, insecurity, unhappiness, as well as spiritual, mental and personality imbalance. These complications result in diminished ability and creative power, serious conflicts, as well as negative emotions as annoyance, jealousy, competition to suppress each other, lack of self-confidence and being ignored (3, 6). Several studies have revealed that sexual dissatisfaction is the primary cause of nearly 80% of marital conflicts, of which 61.4% would later end in divorce (4, 5). However, evaluating and speaking about these problems is usually neglected because of feeling modest, shy, afraid, anxious, embarrassed, inefficient and sinful. These wrong thoughts develop in a way that some women consider "initiating sexual relationship" as losing their chastity and personality. They do not consider the joy of sex as their own right and suffer from having sex (3). These unhealthy relationships between couples are aggravated through tensions and conflicts and will widen the gap between couples, leading to unstable family foundation and increased risk of divorce (6-8).

Moreover, in a number of societies like Iran, marital conflicts and divorce are considered as one of the social inconveniences, which create severe mental tensions. Besides, divorce is not a simple solution for the women, while the husbands who are disrupted mentally and psychologically resist divorcing their wives (4).

Sexuality issues remain a taboo in Iran. Furthermore, according to the records of judicial institutions, the divorce has been increasing at an alarming rate since 2001. Moreover, some studies in this regard showed that sexual dissatisfaction have psychopathology impact on couples and are experienced by many of them who get divorced (4, 9-12). Therefore, performing this study was necessary in order to evaluate prevalence and level of sexual satisfaction in women applying for divorce and to compare it with a control group.

## Materials and Methods

This case-control study included 65 randomly selected women applying for divorce in Isfahan Center of Legal Medicine Organization, Isfahan, Isfahan Province, Iran, during 2011 to 2012. Furthermore, the control group was comprised of 65 randomly selected females from normal population. Age and length of marriage were two factors used for case-matching. This study was approved by Ethic Committee of Legal Medicine Research Centre, Tehran, Iran. All participants also gave an informed written consent. Inclusion criteria were as follow: fertile females older than 18 years old, ability to give informed consent, ability to provide enough information, and being healthy. Exclusion criteria were as follow: chronic medial disease affecting sexual satisfaction, unable to provide enough information, unable to sign informed consent, infertility problem, and females younger than 18 years old.

A female physician helped all participants to fill out a questionnaire designed for this purpose that included two parts: socio-demographic information and factors influencing sexual satisfaction. In order to clarify variables leading to divorce, Larson Inventory of Sexual Satisfaction (ISS) was used to determine sexual satisfaction.

In Iranian population, ISS was previously used as a tool for evaluation of sexual satisfaction with good validity and reliability. The standard questionnaire has 32 items with 16 negative and 16 positive items. Iranian version of ISS with good validity and reliability consists of 25 items (see appendix A) and includes a 5-option Likert scale as follows: never, rarely, sometimes, often, and always (in a 0-4 score range). Based on the scores obtained, each group was placed into four sub-groups: completely satisfied (101-128), relatively satisfied (76-100), slightly satisfied (50-75), and dissatisfied (<50) (12, 13).

Data were gathered without recording names or any other identifying information to keep patient information confidential. Then, data were analyzed using independent t test (for comparing quantitative variables between groups), Chi-square test (for comparing qualitative variables between groups), Fisher’s exact test (for comparing quantitative variables when the anticipated number was <5), and Pearson correlation coefficient (for determining relationship between quantitative variables). All analyses were conducted using the Statistical Package for the Social Sciences (SPSS; SPSS Inc., Chicago, IL, USA) version 16.0 and a p value <0.05 was considered statistically significant.

## Results

Comparison and statistical evaluation of sociodemographic information and factors influencing sexual satisfaction are detailed in table 1.

**Table 1:** Comparison and statistical evaluation of socio-demographic information and factors influencing sexual satisfaction


	Groups			
Parameters	Case	Control	P value	

**Age (Y)**	29.6 ± 6.51	35.31 ± 10.7	0.000	T student
**Age of spouse (Y)**	35.02 ± 7.41	40.74 ± 12.01	0.001	T student
**Age difference between spouses (Y)**	5.42 ± 4.31	5.46 ± 3.6	0.948	T student
**Education level N (%)**				
Illiterate	0	0	0.129	Fisher exact test
First to fifth grade	2(3.1%)	7(10.8%)		
Sixth to eighth grade	7(10.8%)	12(18.5%)		
High school diploma	43 (66.2%)	29(44.6%)		
Associate’s degree	2(3.1%)	1(1.5%)		
Bachelor’s degree	10 (15.4%)	13(20%)		
Post graduate degrees	1(1.5%)	3(4.6%)		
**Education level of spouse N(%)**			0.086	Mann-Whitney
Illiterate	0	0		
First to fifth grade	10 (15.4%)	4(6.2%)		
Sixth to eighth grade	17 (26.2%)	13(20%)		
High school diploma	31 (47.7%)	28(43.1%)		
Associate’s degree	1(1.5%)	2(3.1%)		
Bachelor’s degree	4(6.2%)	13(20%)		
Post graduate degrees	2(3.1%)	5(7.7%)		
**Employment status N (%)**			0.420	Fisher’s exact test
Housewife	44 (67.7%)	47(72.3%)		
Student	5(7.7%)	5(7.7%)		
Worker	1(1.5%)	0		
Employee	7(10.8%)	10(15.4%)		
Unemployed	8(12.3%)	3(4.6%)		
**Employment status of spouse N(%)**			0.193	Fisher’s exact test
Housewife	9(13.8%)	3(4.6%)		
Student	1(1.5%)	0		
Worker	11 (16.9%)	14(21.5%)		
Employee	12 (18.5%)	19(29.2%)		
Unemployed	32 (49.2%)	29(44.6%)		
**Economic status N(%)**			0.004	Mann -Whitney
Good	9(13.8%)	17(26.2%)		
Intermediate	37 (56.9%)	43(66.2%)		
Poor	19 (29.2%)	5(7.7%)		
**Amount of income N(%)**			0.011	Mann -Whitney
High	2(3.1%)	6(9.2%)		
Sufficient	35(53.8%)	46(70.8%)		
Insufficient	28(43.1%)	13(20%)		
**Length of acquaintance prior to marriage (Y)**	9.63 ± 30.16	8.96 ± 32.20	0.906	T student
**Age at time of marriage (Y)**	20.94 ± 4.59	21.56 ± 6.11	0.516	T student
**Age of spouse at time of marriage (Y)**	26.35 ± 5.96	27.02 ± 6.12	0.532	T student
**Duration of marriage (Y)**	8.66 ± 6.45	13.75 ± 13.23	0.006	T student
**Number of children N(%)**			0.010	Mann -Whitney
Zero	25(38.5%)	14(21.5%)		
One	21(32.3%)	14(21.5%)		
Two	15(23.1%)	20(30.8%)		
Three	2(3.1%)	9(13.8%)		
More	2(3.1%)	8(12.3%)		
**Residence N (%)**			1.000	Fisher exact test
City	62(95.4%)	61(93.8%)		
Village	3(4.6%)	4(6.2%)		
**Housing N (%)**			0.000	Fisher exact test
On lease	36(55.4%)	15(23.1%)		
Owned	13(20%)	40(61.5%)		
Her parent’s house	8(12.3%)	3(4.6%)		
Her spouse’s parent’s house	8(12.3%)	6(9.2%)		
Others	0	1(1.5%)		
**Having a special bedroom N(%)**			0.192	Chi-square test
Yes	40(61.5%)	47(72.3%)		
No	25(38.5%)	18(27.7%)		
**Living with other(s) N (%)**			0.120	Fisher exact test
Yes: her parents	13(20%)	4(6.3%)		
Her spouse’s parents	6(9.2%)	7(10.9%)		
Others	2(3.1%)	1(1.6%)		
No	44(67.7%)	52(81.3%)		
**Any types of addiction N (%)**			1.000	Fisher exact test
Yes	0	1 (1.5%)		
No	65(100%)	64(98.5%)		
**Drug abuse by spouse N (%)**			0.000	Chi-square test
Yes	24(36.9%)	1 (1.5%)		
No	41(63.1%)	64(98.5%)		
**Alcohol abuse by spouse N (%)**			0.000	Fisher exact test
Yes	21(32.3%)	0		
No	44(67.7%)	65(100%)		
**Being beaten by spouse N (%)**			0.000	Fisher exact test
Yes	31(47.7%)	0		
No	34(52.3%)	65(100%)		
**Past medical history N (%)**			0.233	Chi-square test
Positive	8 (12.3%)	13(20%)		
Negative	57(87.7%)	52(80%)		
**PH of spouse N (%)**			0.393	Chi-square test
Positive	16(24.6%)	12(18.5%)		
Negative	49(75.4%)	53(81.5%)		
**Sexual problems of spouse N (%)**			0.027	Chi-square test
Positive	9 (13.8%)	2 (3.1%)		
Negative	56(86.2)	63(96.9%)		
**Compulsory marriage N (%)**			0.008	Chi-square test
Yes	13(20%)	3 (4.6%)		
No	52(80%)	62(95.4%)		
**Second marriage of spouse N (%)**			0.000	Fisher exact test
Yes	13(20%)	0		
No	52(80%)	65(100%)		
**Being happy with current partner N (%)**			0.000	Chi-square test
Yes	29(44.6%)	5 (7.7%)		
No	36(55.4%)	60(92.3%)		
Sexual satisfaction score*	55.48 ± 10.14	61.03 ± 13.47	0.009	T student


Data are means ± SD.

As shown, the mean total scores were 55.48 ± 10.14 and 61.03 ± 13.47 in case and control groups, respectively, indicating a significant difference between two groups.

Assessing sexual satisfaction in detail revealed that our cases had experienced lower levels of satisfaction than controls. Notably, the majority percentages in each group were seen in the slightly satisfied level that is opposite to the completely satisfied level from the sixty-five people evaluated in our case group (Table 2). In the case group, 40 (61.5%) and 25 (38.5%) individuals indicated "sexuality worsening" and "without any changes in sexual pattern", respectively, whereas in the control group, 14 (21.5%) and 39 (60%) individuals indicated "sexuality worsening" and "without any changes in sexual pattern", respectively, suggesting significant differences between two groups (p=0.000).

By comparing sexual satisfaction scores with other mentioned factors, we found that having a special bedroom (p=0.037) and having past medical history (PMH) (p=0.011) have significant effects on sexual dissatisfaction.

Significant differences were seen between groups in the following items: "My spouse enjoys having sex with me" (p=0.039), "I have an attractive and exciting sex life" (p=0.002), "My spouse is a good partner for our sexual activities" (p=0.000), "Sexual activity manifolds kind feelings between us" (p=0.001), "My spouse avoids having sex with me" (p=0.005), "Sexual activity with my spouse results in at least one orgasm for me" (p=0.040), "My spouse is very sentimental about my sexual needs and inclinations" (p=0.014), "My spouse cannot make me feel sexually fulfilled after sex" (p=0.004), "I am satisfied with my spouse’s special manner of making love" (p=0.000), "My spouse and I try to find the way to have pleasurable sexual activities" (p=0.008), "I have not been interested in participating sexual activities with my spouse in the preceding months" (p=0.016), "I am satisfied with my sex life" (p=0.000), and "I know myself as a successful person in having sex with my spouse" (p=0.035).

**Table 2:** Frequency distribution of sexual satisfaction within groups


Level of sexual satisfaction	Groups Frequency (percent)		P value
	Control	Case	

**Completely satisfied**	0	0	0
**Relatively satisfied**	7 (10.8%)	2 (3.1%)	
**Slightly satisfied**	48 (73.8%)	43 (66.2%)	
**Dissatisfied**	10 (15.4%)	20 (30.8%)	0.041


## Discussion

Our data demonstrated a decline in divorce rate by longer marital life. Such a decline can be explained that as the years pass, martial satisfaction is to some extent and woman thinks less about sexual activities. As an individual grows older, he/she obtains various experiences and skills for confronting or coping with problems, so individual’s expectations change toward marital satisfaction, This may help couples in overcoming their issues regarding sexual desire, sexual performance, and frequency of sexual activity, while undo the negative effects of confounding items in order to have sexual satisfaction (3).

This is not in accordance with the findings of Shahsiah et al. (13), in which they highlighted the role of sexual satisfaction. They explained that at the beginning of a married life, sexual motivations hide marital concerns, like spousal disputes, blaming each other, economic problem, housing, raising children, etc. However, as time passes, problems have accumulated to the point where marriage starts falling apart.

We found a negative significant relationship between the number of children and divorce rate. Raising children in a family requires wife and husband to spend more time and energy as the result of their additional supportive role as mother and father; therefore, they are subjected to motivations that lead to dangerous behavior and that result in collapse of their marital commitment (3).

Nassimi and Mahdavi showed that there was a positive significant relationship between education and number of children with sexual satisfaction and there was a negative significant relationship between age and sexual satisfaction (14).

Nevertheless, these findings are in disagreement with those of Green et al. and Glazier et al. (15, 16), who explained woman with higher educational level and with monetary independence feels to be useful, whereas the likelihood of divorce and displeasure relating to life will be increased.

Groot et al. (17) suggested that socio-cultural similarities (e.g. the spouse’s age diversity) cause more secure families due to similarities in their life styles and mutual understanding among partner, especially in sexual issues. Their results showed that those with an age difference less than 10 years were more fulfilled with both their marital and sexual life. On the contrary, Litzinger and Gordon (18) found that there is no significant relationship between sexual satisfaction and age difference among spouses.

A consistent or contradictory relationships are affected by factors like education level and age difference between spouses, whereas few studies evaluating the factors like younger age at time of marriage, low education, premarital pregnancy, short premarital acquaintance, personality maladjustment, and low socioeconomic conditions indicated no significant relationships.

Our results showed that divorce rate was significantly more in drug addict spouse. Increasing marital instability due to irresponsible behaviors of individual abusing drug or alcohol in family and society affect marital relationships, indicating relationship between addiction and sexual satisfaction is not significant(4), as shown in this study.

And finally, we tried to evaluate the pivotal role of sexual satisfaction in marital stability of Iranians using a questionnaire; however, participants were likely to respond with considerable bias or to answer in a socially desirable manner. Also, the participants' hesitation to share their private marital relationships should be considered.

Furthermore, we pointed out undesired level of satisfaction seen in our participants as a sample of women living in city of Isfahan Through comprehensive questionnaire and female physician helped us to overcome some of these obstacles. It is impossible to assess the extent to which participants answered truthfully, and the extent to which they prevaricated.

## Conclusion

Our findings suggest that sexual satisfaction plays a pivotal role in marital stability of Iranians. Therefore, development of practical strategies in order to provide cultural intervention is needed to improve Iranian couples’ awareness of their sexual relationship, as well as training in communication skills through their sexual encounters are essential. Since in this study, sexual dissatisfaction was revealed to be an underlying problem leading to divorce, sex education of couples before marriage seems to be of importance. We recommend conducting other prospective studies after education of couples to evaluate the relationship between sexual satisfaction and divorce rate in Iranian population.
